# Rise and fall of the (social) group

**DOI:** 10.1177/03063127221096389

**Published:** 2022-05-29

**Authors:** David Armstrong

**Affiliations:** King’s College London, London, UK

**Keywords:** social groups, group medicine, group therapy, group comparisons

## Abstract

This article maps the rise and fall of the idea of a (social) group across medicine in the context of contemporary analyses in psychology and sociology. This history shows the early 20th century emergence and growth of group medicine, group therapy and group comparisons. In recent decades, however, the idea that groups constituted the basic units of society has been replaced with the emergence of populations and systems that offer a more virtual and abstract context for individual relationships. This has implications for explanation itself as the demise of groups has changed the epistemological ground-rules for understanding identity formation and social change.

In 1974, Ivan Steiner published a well-cited essay entitled ‘Whatever happened to the group in social psychology?’. He claimed that social psychology had become much less ‘groupy’ in the preceding decade than it had been in the 1940s and 1950s.

By the 1960s the group did, indeed, seem to be rather dead, or at least, in very deep hibernation. Its deplorable health or recent demise was sometimes lamented in Annual Review chapters, or over the fourth martini. But the mourners were few in number, and even the immediate family did not seem deeply grieved. (Steiner, 1974: 101)

If, as Steiner claimed, the study of groups had declined in the late 20th century, it was ending an area of research that was barely a century old. Writing at the very end of the 19th century, Simmel had first drawn attention to ‘the persistence of groups’ and the ‘immortality of the group’ as consistent features of human society (Simmel, 1898). For Cooley, too, who later identified the distinction between primary and secondary social groups (Cooley, 1909), it was apparent that ‘just as there is no society or group that is not a collective view of persons, so there is no individual who may not be regarded as a particular view of social groups’ (Cooley, 1902: 3). The first half of the 20th century then witnessed a flurry of interest in studying groups, in part from sociology but mainly from social psychology – though for much of that period the distinction between the two was often unclear.

The history of the word ‘group’ (the group-word) certainly predates the 20th century, but its application in psychology and sociology involved using the term as a descriptor of a new social unit. More than a noun for an aggregate of individuals, the group-word came to imply interaction between a collectivity’s members. This was not simply a ‘technical’ use of the term by these sciences but reflected a more fundamental reconfiguration of the relationships between individuals in the 20^th^ century. This new way of thinking about individuals can be explored by examining the parallel use of the group-word (and its later decline) in clinical medicine, a practical pursuit far removed from the social psychology laboratory or theoretical debates in the social sciences.

The analysis described here is concerned with the routine use of the group-word in discourse and its changing meaning. This calls for a reliance on primary sources as historical accounts of particular groups tend to treat the meaning of the group-word as unproblematic. Use of primary sources in this way also avoids addressing the question of which individuals or organizations (or groups) influenced the spread and changing meaning of the word, a seemingly endless task when language circulates freely and meaning emerges at multiple points. By investigating the parallel trajectories of the group-word across a range of sciences, medical, social and psychological, this article will explore not who said what but how a discourse established what it was possible to say.

The history of (social) groups in sociology and psychology is well-established (Coleman, 1986; McGrath, 1997) and will provide a backdrop for this article’s investigation of the use of the term in medicine. When did it become possible to use the group-word in medical discourse and what meaning did it carry? The strategy employed here is to rely mainly on one source, the *New England Journal of Medicine* (*NEJM*), currently a prestigious journal with a long (and digitized) publication history since 1812. For much of that history (until 1928) it was the *Boston Medical and Surgical Journal*, a provincial US medical journal, at a time when medicine itself was more a regional than international activity. Other sources might reveal a different chronology and some of these, such as the *British Medical Journal* (*BMJ*), are used to complement the main analysis. There seems no reason, however, to believe that use of the group-word, as a component of everyday language, would differ markedly between sources or between regions so long as there was neither explicit encouragement nor dissuasion from using the term in medical writing.

Searches were initially made for the words ‘group’ and ‘groups’ in the *NEJM*, then, as particular contexts were identified, for more specific terms like ‘group therapy’, ‘group medicine’, ‘group dynamics’, and so on. These citations formed the database for the main analysis. Numbers of citations were collected in a spreadsheet to identify broad comparative trends (recognizing that numbers of issues of the studied journals have increased over time), before a more detailed analysis of the historical emergence and decline of groups. Other medical journals were consulted to lend corroborative support as needed. When referencing *NEJM* citations in this article only the year is provided, partly to avoid the final bibliography becoming over-long (a search of the *NEJM* archives using the year and quotation can be used to identify the precise citation) but also to avoid a focus on authorship when the purpose of the analysis is to examine how discourse itself reflects and constructs a social world.

## Classification and connectedness

In the *NEJM* of the early 19th century, there were occasional mentions of a group or groups as applied to things that could be aggregated together. Groups of leeches, of symptoms, of morbid affections, of muscles, and of plants were reported. These collections of objects were based on overt signs of connectedness: ‘There is a grouping of symptoms which the greatest dunce cannot possibly mistake’ (1826). Towards the middle of the 19th century the group label began to be applied sporadically to diseases, especially in relation to disease classification as in ‘a distinct nosological group’ (1854).

Identification of a group of objects implied an underlying classification system but for most of the 19th century the more common descriptor was a class. Marx famously described (economic) classes, restricting comment on ‘groups’ of individuals to descriptions of several workers engaged through the division of labour in an efficient manufacturing unit (echoing Adam Smith’s *Wealth of Nations*). Similarly, the English Registrar-General’s Annual Reports during the 19th century primarily alluded to classes of disease with only occasional use of the term ‘grouped’ to refer to similar diseases presented together in a table. In that sense, identification of an occupational group, say, instead of an occupational class, simply involved replacing one word with a synonym, as in ‘Diseases are ranged in the Registrar-General’s Reports in 112 classes, or we might say groups’ (1860). In 1902 the *NEJM*, for the first time, referred to an age group, to age groups in 1911, and occupational groups in 1919 as the group-word began to replace class.

**Figure 1.**
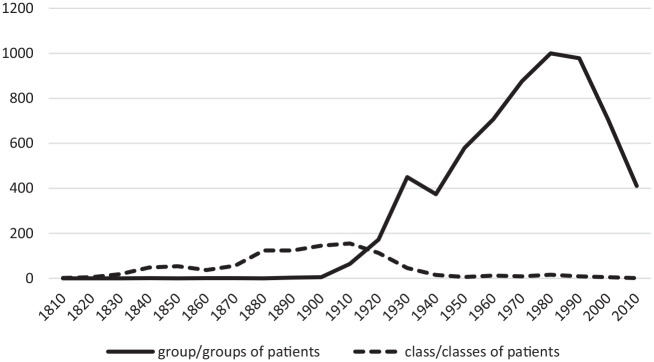
Citations per decade of terms ‘group(s) of patients’ and ‘class(es) of patients’ in *NEJM*.

There were only five mentions of a ‘group of patients’ in the *NEJM* in the 19th century but the term became much more common in the early 20th century (Figure 1 shows the relative decline of class as a descriptor). Similarly, in the *BMJ* there were 16 uses in the last three decades of the 19th century and 87 in the first three of the 20th century. Various types of group were identified in the *NEJM*: There could be a large group of patients or a small one, it could be selected, unselected or consecutive, particular or total, curable or intractable, special or important. In that sense, the group-word was a synonym for class as connectedness was based on resemblance. The identification of groups from the early 20th century, however, began to imply a more dynamic relationship between their members. Groups placed the individual in a local reciprocal relationship based on the aggregate’s defining criteria. ‘The happiness of the social group will be best gained when each individual in the group is happy, and when all are working together for the good of all’ (1915). Indeed, integration into a group was an important factor in mental health: ‘If everyone … could thereby manage these basic forces … in such a way as to fit harmoniously into the social group, there would be no more psychoneuroses’ (1930).

Durkheim’s (1902) *De la Division du Travail Social*, first published in 1893 (though not translated into English until 1933), described the importance of groups but it was the new preface for the second edition issued in 1902 that gave a central role for occupational groups in protecting individuals from the threat of anomie: ‘What we especially see in the occupational group is a moral power capable of containing individual egos [and] of maintaining a spirited sentiment of common solidarity in the consciousness of all the workers’ (1933: 29). Yet for other writers, the group was less an opportunity to protect the individual and more the basis for an indivisible dyad:

Let no one deduce from this that the group is merely the sum total of the individuals which compose it, the net balance of their thoughts and lives. Nothing would be more erroneous. I have already said that laws and processes belong to the group which are foreign to the individual. (Brinton 1902: 26).

Given the reciprocal relationship between group and individual, groups could act jointly but also have a strong effect on group members in their individual actions. The collectivity of patients could ‘judge for itself as an organized social group and as independent members’ their response to campaigns against alcohol consumption’ (1917). Identifying the patient’s social group (a term first appearing in the *NEJM* in 1915) was another factor that ‘may bear on the cause, course, and cure of the disease’ (1925). Correspondingly, social groups could become targets for intervention. Mental hygiene could promote the ‘welfare and sanity of the social group [whether located] in the home, the school, and all agencies for education’ (1918). The efforts of the school physician or industrial physician might ‘not only bring much help to the distressed individual but often to a whole social group’ (1924).

The advent of the group did not foreshadow the demise of individualism but rather a new context and source of dynamism. For Simmel’s path-breaking analysis, the phrase ‘immortality of the group’ implied that ‘The preservation of the identical selfhood of the group through a practically unlimited period gives to the group a significance which, ceteris paribus, is far superior to that of the individual’ (Simmel, 1898: 671). The group depended on the relations of ‘individuals to individuals’; the group was both ‘molecular’, a product of the individuals who composed it, and ‘molar’, in which it acted as a unity. Cooley, too, claimed that groups were a mechanism for enabling a constant experiment in enlarging social experience and in coordinating variety for individuals (Cooley, 1909). In effect, during the last decade of the 19th century and the first of the 20th century a new object emerged that drew together individuals – people and patients – into new connected formations. In this new configuration, groups were no longer collectivities based on similarity – a synonym for classes – but dynamic interacting bodies. As Bonner later noted,

A group exists whenever two or more individuals are aware of one another, when they are in some important way interrelated. In this sense a group is not the same thing as an aggregate of individuals. The latter is a collection, a population, or a class. A group is a number of people in interaction with one another, and it is this interaction process that distinguishes the group from an aggregate. (Bonner 1959: 4)

An awakening medical interest in groups, especially in patient groups and groupings, began to replace a descriptive language of the 19th century with an analytic framework that laid the ground for concepts such as group practice, group therapy, group dynamics, group thinking and group activity. While all these references to groups indicated a new focus on interaction, there seemed to be no direct influences from psychology or sociology (the only time the *NEJM* ever cited the *American Journal of Sociology* was in 1930). More likely, the group-word was in circulation and was used to describe new forms of collectivity in medicine that were also characterized by interaction.

## Group medicine

During the 19th century, medicine had been a solitary activity. Whether visiting patients in their homes or attending the sick in hospital, physicians worked independently as autonomous financial and clinical units. But towards the end of the 19th century, and increasingly in the next, physicians started working collectively. The main vehicle for this coming together was the dispensary or outdoor/out-patient department. In 1900 there were about 100 dispensaries attached to hospitals in the whole of the US but ‘In the last twenty years they have spread like wildfire’ (1922). Unlike the lone physician visiting a patient in a hospital bed:

The dispensary represents an association of specialists, who, meeting in the same building simultaneously, or at times near together, can give to any patient the benefit of joint consultation. The medical men [sic] themselves also profit greatly in thus learning from one another. (1914)

As outpatient departments grew in size and extent they became differentiated by specialist area, which brought together surgeons with other surgeons, cardiologists with other cardiologists: ‘The medical principle on which the dispensary is founded is that of the organization of specialties, … a function which is likely to play a large part in the medical practice of the future’ (1914). Once the structuring of finances was resolved the ‘group medicine system’ enabled the patient to have ‘the knowledge of many specialists for one fee’ (1917).

Beyond the growing out-patient departments, physicians, like their patients, were also coalescing into groups. It became opportune to explore the value of being a collective and to discuss ‘the benefits and dangers of group practice’ (1921). Several explanations were advanced for this new phenomenon. Perhaps it was simply the case that ‘The habits and fashions of the age have engulfed the doctor and he has been drawn away from the country from home practice to specialization, to office, to hospital, to group practice’ (1921). Or perhaps it was so patients could benefit from the collective expertise of several clinicians: ‘It is to meet the need in these cases (where the diagnosis is doubtful or obscure) that the idea of group practice has sprung up’ (1922).

Despite the rapid growth of group practice, there were still concerns: ‘group practice is still regarded as in the experimental stage and subject to the dangers of certain evils, regardless of its unquestioned merits [but] modern medicine can certainly be practiced most effectively by a group system’ (1925). The founder of the Mayo Clinic had to defend himself against accusations of being ‘the father of group medicine’ (1927). Much of the resistance to group practice identified the threat of commercialism ‘using the methods of big business with its boosting, financial programs, credit, sales appeal, etc’ (1934). Yet group practice was everywhere expanding:

Group practice has developed on hospital and clinic staffs and in private groups. The general practitioner can and should be associated with groups along with the specialists, for his own benefit as well as for that of his patients. The efficiencies and economies of grouping are bound to win out in the long run. (1934)

If medical groups began to proliferate after World War I, there was equal evidence ‘that a significant expansion of group practice is also following World War II’ (1947). By 1954 it was reported that ‘The number of group-practice units and the number of physicians associated with this form of practice are both gradually increasing’ (1954). How group practices worked became a subject of scientific study (1947). The Group Health Association reported its experience between 1938 and 1947 as accusations of being a form of ‘socialized medicine’ were replaced by an appreciation of the value of group practice (1949). ‘Group medical practice is here to stay’ (1962).

During the late 1950s and early 1960s the sociology of the professions – using medicine as the archetypal occupation – began to emerge as a specialist area. The cornerstone of the profession was identified as its sense of community (Goode, 1957). Clinical medicine did not involve independent physicians carrying out solo practice but coalescing groups of practitioners – some, hundreds strong – who were conducting the activity of ‘doctoring together’, to use Freidson’s (1975) phrase. How better to describe contemporary medicine than as a ‘colleague group’ (Freidson, 1970; Freidson and Lorber, 1972)? Yet for all these attempts to identify the defining characteristics of professionalism, the medical profession as a group, as an interacting community, was in fact only a few decades old.

## Group therapy

A novel method of managing patients with tuberculosis was reported in 1910. The innovation involved enrolling patients into Suburban Tuberculosis Classes: ‘Here, by means of weekly meetings of the class, home records of pulse, temperature and daily life, and visits in the homes by our nurse, we have been able to keep our patients under strict supervision’ (1910). The class method increased the therapeutic effect of clinical intervention as ‘patients often instruct and encourage each other by testifying convincingly of their own improvement’ (1920). One small group ‘frequently arouses the interest of a whole neighborhood by providing a graphic illustration’ (1924). In the group ‘lies the opportunity to best develop the art of learning how to live through competitive projects, through class loyalty and many other factors allied with the group method’ (1927). The concurrent grouping of doctors and patients into specialist clinics enabled the spread of this approach as ‘in many instances class methods have been found most effective’ (1920).

A lecture method to groups of patients had been tested in psychiatry in 1920 when ‘a surprisingly high percentage of remissions among the psychotics, and a still higher percentage of cures among the more severe psychoneurotics were reported’; later, experiments ‘utilized the hospital loud-speaker system for delivering lectures in therapeutics’ (1939). In 1930, ‘the first thought-control class dedicated to the purpose of re-forming the emotional habits of the psycho-neurotic’ was inaugurated; the approach employed was described as ‘childish in its appeal, thus imitating the usual responses of such patients’ (1939).

Several classes are held each week and any new members are given front-row seats, where they command some attention. Older members are urged to make friends with these novices, and thus an increase in social intercourse is afforded both patients. (1940)

Group psychotherapy seemed valuable for patients with mild psychoneurosis and therefore proved ‘an effective filter that would adequately take care of the less severe cases and refer the refractive patients to a special psychiatrist’ (1940). Indeed, the group itself was seen to have therapeutic benefit as its influence alone could correct emotional disorders: ‘An advantage of group therapy is the feeling of security and relief for the individual in finding that he is not alone in having thoughts and impulses which have seemed to him to isolate him from society’ (1940).

After WWII, experiments were held with ‘leaderless groups’ in which patients selected their leader from their members. More importantly, therapists began to distinguish between ‘treating individuals in a group and true group psychotherapy, in which treatment is largely through the interaction of members of the group upon each other’ (1948). In that sense the group was more than an aggregate sharing some common attribute, but one that transformed its members and reinforced their connectedness: ‘true group psychotherapy [is when] … the interaction of the group, the psychologic effect of members upon each other, is the therapeutic agent’ (1948).

Group psychotherapy was based on – and promoted – a social model of mental illness. Symptoms were traced to the ‘relationship of the individual to his manner of functioning in social situations — i.e., in the type and quality of his “connectedness” to the groups which make up his life space’ (1973). The presence of the group and its interactions offered a ‘transitional social system’ prior to reintegration into the home community on discharge (1973). The group functioned to re-motivate and re-socialize patients back into their communities. ‘Open Psychiatric hospitals’ with a therapeutic climate designed to foster group efforts seemed the best way to return many long-term patients to their communities (1963). In place of the exclusionary policies of the old asylum, there was ‘group identification, pride of accomplishment, and an opportunity to develop and participate in the group process of rehabilitation’ (1963).

Group therapy also extended to physical illness, especially where there was believed to be an emotional component. Group methods could help in regulating dietary regimens for patients with peptic ulcer, with the adjustment of hypertensive patients, with blind patients, with undernourished children, diabetic patients, ante-partum and post-partum women and people requiring corrective exercise, with ileostomy patients and in multiple sclerosis (1955). It could be used ‘in social groups, in the armed forces and even in the prisons, touching the fields of physician, psychiatrist, psychologist, social-service worker and chaplain’ (1955). While group therapy might have had minimal effect on physical illness, it offered a practical method of facilitating emotional adjustment that might in its turn lessen the intensity of symptoms. Above all, it was the way in which group therapy satisfied the patient’s ‘need to belong, through intragroup support, by consistent clarification of medical aspects and through the interest of the physicians’ (1957).

During the 1930s and 1940s, group interactions also came more to the attention of psychologists. Lewin, for example, published numerous papers between 1935 and 1946 on group dynamics (Lewin, 1948) while Bales reported on interaction in small-decision-making groups with what he called ‘interaction process analysis’ (Bales, 1950). This involved studying social interaction in small face-to-face groups often using two-way mirrors. In the 1940s, Bion brought a psychoanalytic perspective to bear on group dynamics (Bion, 1961) while Rogers promoted person-centredness in the interactions of ‘encounter groups’ (Rogers, 1970). A group was ‘in a continuous process of restructuring, adjusting, and readjusting members to one another for the purpose of reducing the tensions, eliminating the conflicts, and solving the problems which its members have in common’ (Bonner, 1959: 5–6).

The internal dynamics of groups established the reciprocal relationship between the individual and the collective.

A democratic society derives its strength from the effective functioning of the multitude of groups which it contains. Its most valuable resources are the groups of people found in its homes, communities, schools, churches, business concerns, union halls, and various branches of government. (Cartwright and Zander, 1953: ix).

The corollary was that knowledge of groups and group methods became a skill needed by many occupations, from supervisory staff and public welfare agencies through rehabilitation and correctional services to mental and public health (Abrahamson, 1959).

## Group comparisons

For most of the 19th century, the basic design of an experiment was to offer an intervention, ideally under controlled conditions, and then observe its effects. This procedure could be conducted on animals in the laboratory or patients in hospital beds. The new 20th century discourse on groups offered the possibility of a new experimental design: the comparison of two or more groups. Group comparisons could reveal prognostic signs, as in, ‘We decided to compare these two groups of patients to see if we could determine what symptoms could be considered favorable, or unfavorable, in forecasting a prognosis’ (1912) or diagnostic categories, when ‘The possibility was made out that there are two groups of dementia precox cases, a group subject to early death … and a far more viable group’ (1913).

Comparing groups addressed the vexed problem of individual variability. By studying groups of individuals, averages, together with some measure of dispersion, could be determined: ‘This made it possible to attack the whole problem from a different angle’ (1921). The death rates from alcohol consumption, for example, could be compared for groups of ‘moderate or immoderate drinkers’ (1904). Establishing norms for different groups also became an important objective in group comparisons. A study in 1927, for example, examined whether there were ‘any characteristic variations from normal in the basal metabolic rate in the three different groups of arthritis’ (1927). The natural history of a disease could also be explored by establishing different baseline groups and following them up: ‘The whole group of cardiac patients was then classified according to symptoms to see if the anatomical changes found, or the duration of life, varied in the different groups’ (1930).

But perhaps most significantly, group comparison methods provided a novel way of testing the effectiveness of medical treatments. This approach to group comparisons did not involve different groups but similar ones that received different treatments with a view to assessing their value. Comparisons of two or more groups offered a new way of evaluating medical interventions and established the basis for the late 20th century ascendency of clinical trials and evidence-based medicine (Smith and Rennie, 2014).

Assembling similar groups, however, was not easy: ‘Although accurate comparisons are impossible, it seems that patients, who are relieved, are different in character in the different groups’ (1913). One solution was to have a control group, a term first used in the *NEJM* in 1916 (and first used in the *BMJ* in 1904). Between 1920 and 1950 there were over 250 mentions of control groups in the *NEJM*, as it increasingly became a key component of research design. From the serological treatment of lobar pneumonia (1924) and the effect of cod liver oil on rickets (1924) to the effect of orange juice on height (1927) and the prevention of cold by vaccine therapy (1931), control groups provided the essential comparator for judging the value of treatment. Indeed, towards the end of the inter-war years, many of the citations to a control group were either critical of its absence or apologetic for its inadequacy.

More attention began to be paid to methods for choosing control groups, the idea being that both experimental and control groups should be as similar as possible if valid inferences about intervention effectiveness were to be drawn. In 1935, a ‘control group of 25 infants picked at random from clinics’ was described and, in the same year, a control group of ‘alternate cases’. In 1952, a randomization technique was described based on whether the last digit of a patient number was odd or even to assign to intervention and control groups. By the late 1950s the advantages of randomized assignment to groups had become apparent, though two decades later a critical article could still point out that only 12% of clinical trials of new anti-cancer agents used randomization: ‘How ironic that treatment designed to contain the disease most feared, publicized, and politicized in the United States should so often have been denied the benefit of what many regard as the only sound method of clinical evaluation’ (1972).

At the same time as control groups established their dominance in treatment evaluations, psychologists and sociologists identified the importance of the reference group, a term first coined in 1942, to capture the relation between an individual and their perception of the group to which they belonged. Reference group theory, developed particularly by Merton and his colleagues (Merton and Kitt, 1950; Merton and Rossi, 1957), provided the benchmark and contrast needed for comparison and evaluation of group and personal characteristics. Reference groups underpinned social comparison theory that claimed that individuals needed to affirm their own opinions and self-evaluations by comparing themselves to others. Comparisons between groups could therefore work in a twofold way. First, group could be compared with group, as in medicine, to enable evaluations of clinical interventions, particularly in the context of control groups. And secondly, groups provided the comparator for individuals to evaluate themselves. After WWII, reference group theory provided the foundation for a large body of research in sociology on race relations, worker job satisfaction, and mass communication (Pepitone, 1981).

The development of reference group theory reflected the meaning attached to group affiliation. On the one hand, group membership might explain why some patients ‘evaluate any deviation from the normal in terms of nonconformity with accepted patterns of behavior rather than in medical or physical terms’ (1948). ‘A careful interview by the physician’, for instance, ‘will disclose that the patient belongs to a social group that admires addiction’ (1964). In-groups and out-groups therefore became mechanisms for evaluating social position. Such qualitative comparisons underpinned social science constructs from reference groups to stigma (Goffman, 1963). And given that society was no more than a collection of many groups, the group began to provide the moral compass for community beliefs and behaviour and the basis for ethical debates: ‘Do individuals, groups and planetary communities have the right to refuse to conform with beneficial health practices? Does a society or social group have the warrant to impose its values’ (1975).

Comparison, control and reference groups brought people together in new configurations. Whereas group practice and group therapy had involved direct interaction between group members, group comparisons involved a virtual juxtaposition. In the 19th century, mortality could be compared by occupational classes, but these classes were constituted by a wider classification system of all occupations. The new approach to group comparisons was as if samples were taken from classes based on some defining characteristics. The new groups were virtual, constructed in an ad hoc way by identifying a single characteristic for group membership. If two groups were identified for comparison it was not because they belonged to a wider classification system but because they had specific characteristics in common. Such characteristics were likely only a single attribute of a person, their blood pressure or height, say, that allowed them to be assigned to groups. Members of a comparison group or reference group might never meet but their existence provided the referent to constitute a group with shared characteristics. Such virtual groups did not depend on physical or social proximity but upon similarity and difference. In part they resembled the connectedness of classes or cells in a classification table but they differed in that their very existence was predicated on direct group comparison and difference.

The origins and characteristics of group comparisons in developing ‘fair trials’ of medical treatments is now the subject of an extensive literature, much of it collated by the James Lind Library (Chalmers and Abbasi, 2020). But what is of interest in the present context is not the ‘successes’ of randomization or control groups, nor the role of individuals in these achievements, but rather how the identification of groups from early in the 20th century fit into a broader pattern of new ways of apprehending collectivities. Yet, just as the idea of the group achieved so much in terms of understanding society and its practical organization, research methodologies and clinical therapies, moral ethics and psychological identity, it began a period of significant decline.

## Decline of the group

According to McGrath, ‘group research suffered a system crash within North American social psychology in the late sixties and early seventies’ (McGrath, 1997: 7). Hogg and Tindale concurred, observing that ‘by the mid-1980s, the notion of groups as a central focus in the ﬁeld had all but evaporated’ (Hogg and Tindale, 2001: ix). The earlier conception of the group as a unit of several interacting individuals occupying ‘real space’ (Shaw, 1981) was undermined by post-war social cognition models that internalized the idea of a group. A ‘born-again group’ was simply a social representation, a figment of the mind: Instead of the individual being in the group, ‘the group was now within the individual’ (Hogg and Abrams, 1988: 19). Cognitive dissonance, attribution and social identity theory relocated groups into the mind of the individual: ‘groups do not exist unless they are perceived to exist by the membership … for “groupness” is essentially an attribute projected onto the social world by the individual’ (Gergen, 1989: 465).

In sociology, too, the high point of the group was the middle of the 20^th^ century: ‘Fundamentally, the role of the sociologist working in social and personal disorganization should be not only analysis of formal and informal group structures but of the relation of the person to social groups’ (Clinard, 1949: 257), a position echoed in Homan’s (1950) classic, *The Human Group*. But then, there was what Coleman described as a ‘watershed’ when survey research of populations began to replace ‘community studies’, with a shift in the unit of analysis from the group to individuals who were ‘“independently drawn” members of the population’ (Coleman, 1986: 1315).

The ‘evaporation’ of the group across the social sciences was matched by a decline in medicine, as indicated by discourse in the *NEJM*. As Figure 1 shows, the basic descriptor of ‘group(s) of patients’ started declining from the 1980s. From the 1980s, interest in group practice also decreased rapidly while there was hardly any reference to group medicine. The *BMJ* showed a similar pattern, as mention of ‘group of patients’ halved between the 1980s and 1990s and then halved again in the following decade. Reference to group medicine and group practice in the *BMJ* also started declining in the 1980s.

The decline in mentions of group therapy was neither so precipitous nor consistent. For some journals that had taken a major interest in the field the decline followed the pattern of group medicine and group practice. While the *NEJM* maintained interest in the field until the 1990s, for the *American Journal of Psychiatry* most mentions were in the 1970s before declining by more than half by the 1990s. Similarly, the *BMJ*’s highpoint was in the 1960s, before beginning a rapid decline. In the early 21st century, however, there was a revival of interest in certain, mainly clinical, psychology journals. In part this was driven by a growing interest in using cognitive behaviour therapy (CBT) in groups. Yet, the main justification for group CBT was the efficiency of treating several patients at the same time even though individual therapy remained the benchmark of effectiveness (Hollon and Shaw, 1979). The other difference between the therapeutic group of the mid-20th century and that of the early 21st century was the site of intervention. For the earlier model, the group itself delivered therapy whereas in CBT groups the main therapeutic work had still to be carried out by individuals outside the group setting.

The other successor of the dynamic group of the early post-war decades was the team. The first task in defining teamwork seemed to be to differentiate a group from a team in terms of their goals:

Groups become teams through disciplined action. They shape a common purpose, agree on performance goals, define a common working approach, develop high levels of complementary skills, and hold themselves mutually accountable for results. (Katzenbach and Smith, 1993: 14)

A team was more than a group, more than a mini-social unit, but a collective working together on some external task: ‘Teams are more productive than groups that have no clear performance objectives because their members are committed to deliver tangible performance results’ (p. 15). Outputs and productivity became the new metric for understanding group/team interaction, generating research questions more concerned with the dynamics between rather than within groups (Sanna and Parks, 1997).

In medicine, reference to teams and teamwork also increased in the closing decades of the 20th century – descriptions of the ‘clinical team’, for example, first appeared in the 1970s in the *NEJM* (mention of teams in the 19th century referred to horse and wagon combinations). The clinical team was both more and less than a group. On the one hand, team members might work alone: ‘Continuity may be provided by a clinical team, perhaps more effectively than by one clinician’ (1997) and on the other hand the team provided an efficient division of labour involving teams ‘of investigators with different skills and backgrounds’ (2003).

The trajectory of control and comparison groups showed a diverse pattern. While the number of mentions of control group in the *BMJ* halved between the 1980s and the last decade of the century, in the *NEJM* they continued to grow. But while the words remained the same, former members of groups with characteristics in common were reconstrued as being elements of bigger (mostly virtual) populations. The contemporary rise of the confidence interval in statistics from the mid-1980s, for instance, established an inferential link between the sample and the population from which it was supposedly drawn (Armstrong, 2017).

**Figure 2.**
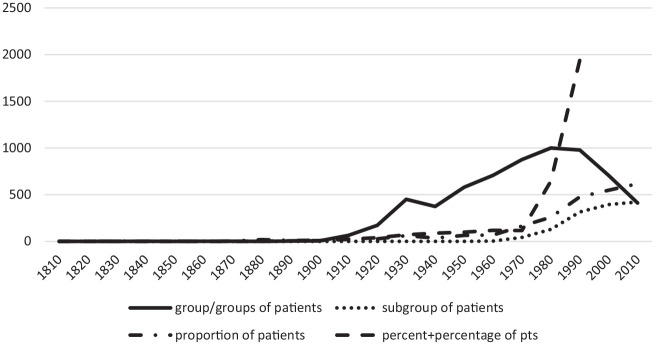
Comparative decline in citations per decade of term ‘group(s) of patients’ and increase in alternative constructs in *NEJM.*

This shift from groups to population samples can be seen in the emergence of alternative constructs in the last two decades of the 20th century (see Figure 2). Alluding to ‘percent’ and ‘percentage’ of patients, for example, became much more common, a meaningless statistic in the context of an indeterminate sized group. Reference to proportion and sub-group of patients also increased as group(s) of patients declined. Together, these new terms pointed to a new ‘whole’, perhaps a population or an implicit population. But these were virtual, assemblages of patients with some common characteristics but no necessary knowledge of each other.

If the group as a simple collectivity, as a classificatory device, was in part replaced by populations and samples, the interacting group was replaced by systems and networks. Since the 19^th^ century the latter had referred almost exclusively to biological entities (such as nervous system or capillary networks) but from the second half of the 20th century, clinical work was delivered through what were increasingly referred to as medical or health care systems. Medical autonomy that had been protected by working together in groups could now face the fact that ‘independence in the present interdependent medical-care system is illusory’; the professional role in primary care, for instance, was ‘system-defined’ (1971). Care networks, hospital networks, welfare networks and communication networks began to replace group medicine as the analytic frame for health care provision. Patients too became members of systems. The ‘family system’, for example, that earlier in the century had referred to the ‘boarding out’ of mental health patients, became a new locus of therapy: ‘the study of the family is necessary in all cases of psychopathology since the presenting patient may be only the symptom of a disturbed family system’ (1971).

Some use of the group-word continued such as in socio-economic or racial or ethnic group. But these were not co-located and interacting groups but might better be described as populations rather than aggregates occupying ‘real space’. Similarly, terms such as reference group began to apply to statistical rather than social comparators. One new form of interacting group that did emerge was the methodological innovation of the focus group (from the 1970s). This involved a group interview that regarded interactions between participants as an important part of the method. Yet these were ephemeral groups, held together for a short time period, a shadow of the former group ‘persistence’.

## The rise of (virtual) populations, systems and networks

According to the above analysis, for psychology, sociology and medicine, the group-word had an early 20th century origin and later decline. This conclusion, however, is based on a limited number of sources and it is possible that other journals or texts were using the group-word routinely before the 20th century. Equally, the group-word may have persisted in some other areas. Some corroboration for a pattern of rise and fall, however, is provided by Google Books Ngram Viewer which searches over 5 million books (Michel et al., 2011). This shows a sharp increase in the rate of use of the word ‘group’ during the early 20th century before a decline in the last few decades suggesting deployment of the word in the sciences described above reflected its use across language in general. A similar picture is presented by mapping the terms ‘group practice’, ‘group therapy’, ‘group comparisons’, ‘psychological … group’ and ‘sociological … group’ (using dependencies) in the English corpus.

It is possible that the rise and decline of the group-word may just reflect a fashion, a case of one synonym replacing another in popular language. But earlier descriptors covered in this article, such as classes, and later ones such as populations or networks, carry different connotations. Further, the group-word emerged in seemingly independent ways to describe very different phenomena that had one common theme – a collectivity of potentially interacting individuals. It seems unlikely that experiments in the psychology laboratory informed the drive towards groups practice in medicine or the decisions to treat patients in groups earlier in the century inspired the later analysis of group dynamics by one-way-mirrors. The common pattern of rise and fall in language therefore suggests a fundamental shift in the relationship of the individual to the collective over the last century.

Of course, once the group-word was in routine use it was possible to apply it to human aggregates in any past period, well before the late 19th century. That would enable histories of groups of doctors, or groups of patients, or of specific types of group, to be identified over several centuries. As Cartwright and Zander noted, ‘The literature about groups goes back to the distant past’ (1953: ix) or as Greco (1950) observed, ‘as Francis Bacon, Hugo Grotius, Darwin and Kropotkin had noted, group life is as old as individual life’ (p. 2). But such accounts could not have pre-dated the emergence of the group-word and in that sense these groups in history belong to a 20th century discourse.

The new emphasis on populations and their sub-divisions marked a change in the dynamic between the individual and the aggregate. The wider society had been construed as a group phenomenon, as ‘a constellation of social groupings’ (1952); groups had both contained and constructed individual identity: individuals had been ‘associated into overlapping groups for trade, occupation, religion and amusement’ (1952). The mere presence of others implied changes in the individual to the extent that the group-mind was more than a metaphor for many inter-war psychologists. In contrast, there was no necessary interpersonal relationship of the individual to the new population, sample or proportion. Individuals could be counted and aggregated but those accumulations were post hoc, gathered together because of some similarity or other. No longer could group structure, culture or opinion directly influence the individual, nor could individuals be summed and joined as a group identity especially when submerged in team tasks. With the new emphasis on cognitions, the group was located within the individual so that when some collectivity was established, perhaps for an experiment or a clinical trial, the individuals concerned would be strangers to each other: ‘[T]hey were people with whom the individuals had never interacted in the past, were not interacting with in the present, and did not expect to interact with in the future’ (Berscheid, 1999: 262). As Danziger noted, ‘actual social groups were gradually replaced by hypothetical groups that had a purely statistical reality’ (Danziger, 2000: 345).

Psychologists speculated that the demise of groups was a product of the wider social milieu and its sense of stability – though predictions of a revival of interest in groups with increasing social conflict were not realised (Steiner, 1986). Danziger claimed the advent of randomization had constructed artificial groups that had no interpersonal connections (Danziger, 2000: 345); but this would not account for the concurrent decline of interest in, say, group medicine or group therapy. The problem is that shifts from worlds dominated by classes to groups and then to populations and systems also mark changes in the nature and legitimacy of explanation itself. Groups provided the epistemological ground-rules for explanation, from individual behaviour to theories of wider social change. Individual identity was predicated on group membership; the group-mind jostled with the individual mind in explaining human behaviour. Social groups constituted the building blocks of social order and social change. But in a ‘post-group’ world, individuals are members of virtual populations, assignment is based on individual characteristics, and they operate in complex systems of which they might have little awareness. Populations exert no collective agency as members might never have met each other, even less interacted, while systems, which might enable or constrain, are more than the sum of individuals.

In summary, the way in which social aggregates are perceived plays an important role in defining the individuals who belong to them. In the 19th century, individuals were seen through the grid of a classification system that divided things and people into classes. In the early 20th century, a class began to be replaced by a group, but gradually the group took on a new role. Whereas a class was simply assigned, a group marked a new dynamic, an interactive identity for individuals whether in psychological theory or clinical practice. The replacement of groups by populations, population samples, systems, networks and team tasks in the late 20th century therefore marks another shift in perception across medicine and the social sciences, about what can be said, thought and acted upon, and what cannot.
